# Dl‐3‐n‐butylphthalide promotes synaptic plasticity by activating the Akt/ERK signaling pathway and reduces the blood–brain barrier leakage by inhibiting the HIF‐1α/MMP signaling pathway in vascular dementia model mice

**DOI:** 10.1111/cns.14112

**Published:** 2023-02-08

**Authors:** Ping Che, Juan Zhang, Mingqian Yu, Ping Tang, Yanhui Wang, Aolei Lin, Jing Xu, Nan Zhang

**Affiliations:** ^1^ Department of Neurology Tianjin Neurological Institute, Tianjin Medical University General Hospital Tianjin China; ^2^ Department of Neurology Gucheng Hospital in Hebei Province Hengshui China; ^3^ School of Medicine Nankai University Tianjin China; ^4^ Department of Neurology Tianjin Medical University General Hospital Airport Site Tianjin China

**Keywords:** bilateral common carotid artery stenosis, blood–brain barrier, DL‐3‐n‐butylphthalide, synaptic plasticity, vascular dementia

## Abstract

**Aims:**

DL‐3‐n‐butylphthalide (NBP) exerts beneficial effects on global cognitive functions, but the underlying molecular mechanisms are still poorly understood. The present study aimed to investigate whether NBP mediates synaptic plasticity and blood–brain barrier (BBB) function, which play a pivotal role in the pathogenesis of vascular dementia (VaD), in a mouse model of bilateral common carotid artery stenosis (BCAS).

**Methods:**

NBP was administered to model mice at a dose of 80 mg/kg by gavage for 28 days after surgery. Cognitive function was evaluated by behavioral tests, and hippocampal synaptic plasticity was evaluated by in vivo electrophysiological recording. Cerebral blood flow (CBF), hippocampal volume, and white matter integrity were measured with laser speckle imaging (LSI) and MRI. In addition, BBB leakage and the expression of proteins related to the Akt/ERK and HIF‐1α/MMP signaling pathways were assessed by biochemical assays.

**Results:**

NBP treatment alleviated cognitive impairment, hippocampal atrophy, and synaptic plasticity impairment induced by BCAS. In addition, NBP treatment increased CBF, promoted white matter integrity, and decreased BBB leakage. Regarding the molecular mechanisms, in mice  with BCAS, NBP may activate the Akt/ERK signaling pathway, which upregulates the expression of synapse‐associated proteins, and it may also inhibit the HIF‐1α/MMP signaling pathway, thereby increasing the expression of tight junction (TJ) proteins.

**Conclusion:**

In conclusion, our results demonstrated the therapeutic effects of NBP in improving cognitive function via a wide range of targets in mice subjected to BCAS.

## Graphical abstract

DL‐3‐n‐butylphthalide may improve cognitive function via mediating the Akt/ERK and HIF‐1α/MMP signaling pathways in a mouse model of bilateral common carotid artery stenosis.

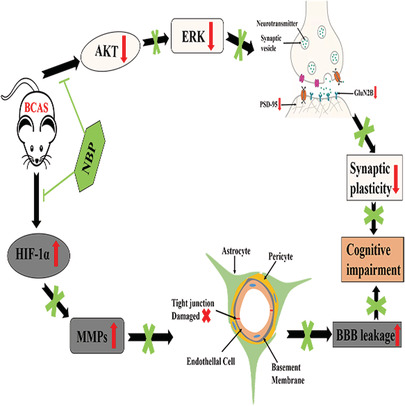

## 1 INTRODUCTION

The pathogenic and pathophysiological mechanisms of vascular dementia (VaD) are complex and include hypoperfusion, endothelial dysfunction, neuroinflammation, blood–brain barrier (BBB) destruction, white matter demyelination, and impairment of synaptic plasticity.^1^ Recent studies have suggested that enhancing synaptic plasticity and protecting BBB integrity are promising strategies for VaD treatment.^2^, ^3^, ^4^

Synaptic function in the hippocampus is fundamental for the formation and maintenance of memories. N‐methyl‐D‐aspartate (NMDA) receptors located in the postsynaptic membrane can be activated by excitatory neurotransmitters in the central nervous system and trigger a series of actions that induce the generation of long‐term potentiation (LTP).^5^ Pathological changes in synaptic structural and functional plasticity are common in Alzheimer's disease (AD). VaD might have similar and common pathophysiological processes as AD since several common vascular risk factors, such as coronary heart disease, cardiac arrhythmia, hypertension, cerebrovascular accident, obesity, smoking, and physical inactivity, have been observed in both diseases.^6^ Lower levels of NMDA receptors and postsynaptic density protein 95 (PSD‐95) in the hippocampus accompanied by impairment of synaptic plasticity and memory were previously observed in AD mouse models.^7^, ^8^ Moreover, regulation of LTP and the expression of NMDA receptors, especially the GluN2B subunit, in the hippocampus was found to be closely related to the alleviation of learning and memory impairment in a rat model of VaD induced by bilateral occlusion of the common carotid arteries (2VO) in our previous studies.^9^, ^10^ The Akt (protein kinase B, PKB) signaling pathway has a significant impact on synaptic plasticity and neurotransmission and a myriad of related brain functions, such as learning and memory.^11^, ^12^, ^13^ Extracellular signal regulated kinase (ERK) is a member of the mitogen‐activated protein kinase (MAPK) family, which is also critical for synaptic plasticity and memory formation.^14^

The BBB acts as a shield and plays a critical role in maintaining central nervous system homeostasis, such as by preventing potential blood‐borne toxins from entering the brain and maintaining brain metabolic balance.^15^ An increasing number of studies have indicated that interventions that reduce BBB leakage can help prevent dementia.^16^, ^17^, ^18^ Hypoxia‐inducible factor alpha subunit (HIF‐1α) affects signaling pathways associated with development, metabolism, inflammation, and complex physiological processes.^19^ A previous study showed that an HIF‐1α inhibitor increased survival and improved BBB function in *S. pneumonia‐*infected mice, suggesting that HIF‐1α plays a pivotal role in maintaining the integrity of the BBB.^20^ Matrix metallopeptidases (MMPs), particularly MMP‐9 and MMP‐2, can lyse the extracellular matrix and degrade tight junctions and basal lamina between endothelial cells,^21^ which are essential for maintaining the BBB.^22^ Furthermore, it was demonstrated that the accumulation of HIF‐1α can increase the transcription and activity of MMP‐9/MMP‐2 and downregulate the expression of TJ proteins in human epidermal keratinocytes exposed to metal nanoparticles.^23^ The HIF‐1α/MMP‐9 signaling pathway also plays a central role in inhibiting the expression of TJ proteins and disrupting the BBB in a traumatic brain injury mouse model.^24^

DL‐3‐n‐butylphthalide (NBP) has been approved and widely used in the treatment of ischemic cerebrovascular diseases in China, especially in patients with acute ischemic stroke. It was further demonstrated that NBP improved global cognitive functions in patients with subcortical vascular cognitive impairment without dementia in a multicenter, randomized, double‐blind, placebo‐controlled trial in China.^25^ Several studies have investigated the ability of NBP to ameliorate neurological injuries in various animal models of VaD,^26^, ^27^ suggesting that it has multiple effects, including enhancing synaptic plasticity^28^ and maintaining BBB integrity.^27^ However, it is unclear whether these effects of NBP on the BBB and synaptic function are mediated by the HIF‐1α/MMP and Akt/ERK signaling pathways, respectively, in VaD. In the present study, we aimed to explore the molecular mechanisms by which NBP alleviated cognitive impairment by enhancing synaptic plasticity and reducing BBB leakage in depth in a novel VaD mouse model established by bilateral common carotid artery stenosis (BCAS).

## 2 METHODS

### 2.1 Animals

Male C57/BL6 mice weighing 20–24 g (8–10 weeks old) were purchased from Beijing Vital River Laboratory Animal Technology Co., Ltd. All animal experiments were approved by the Animal Care Use Committee of Tianjin Medical University General Hospital, and the experimental procedures were performed in accordance with the Animal Management Rules of the Ministry of Health of the People's Republic of China.

### 2.2 The BCAS model and experimental groups

All mice (*n* = 80) were randomly assigned to the SHAM group, the BCAS group, the BCAS + vehicle group, or the BCAS + NBP group, with 20 mice in each group. The BCAS operation was performed according to the description in previous studies.^29^ In brief, both common carotid arteries (CCAs) were exposed, and then a loop of the microcoil was installed below the carotid bifurcation. Half an hour later, another microcoil was applied to the contralateral CCA via the same procedure. Mice in the sham group were subjected to the same operation procedure, except application of microcoils to the CCAs.

NBP (formula: C12H14O2; purity >95%) was provided by Shijiazhuang Pharmaceutical Co. Ltd. NBP was diluted with vegetable oil to generate a stock solution. The BCAS + NBP group was treated with NBP (80 mg/kg) by gavage once a day for 28 days beginning the third day after surgery. The BCAS + vehicle group received the same amount of vegetable oil by gavage.

### 2.3 Behavioral tests

#### 2.3.1 Open‐field test

At day 28 after NBP treatment, the open‐field (OF) test was used to evaluate the activity and anxiety‐like behavior of the mice according to a previously reported procedure.^30^ The mice were placed in an open arena (45 cm × 45 cm × 45 cm), which was partitioned into 25 (5 × 5) equal‐size squares, with the middle nine grids (3 × 3) being considered the central zone. The total distance traveled, crossing times, and total duration in the central zone were recorded for 5 min.

#### 2.3.2 Novel object recognition test

After the OF test, the mice underwent the novel object recognition (NOR) test to assess memory as previously reported with a few modifications.^31^ Exploratory preference, which was defined as the discrimination index (DI) ((time spent exploring the novel object/total time spent exploring both objects) × 100), was calculated for analysis.

#### 2.3.3 Y maze test

The Y maze test was conducted after the NOR test on the same day. The mice were placed at the end of one arm and allowed to move freely for 8 min, and their movement trajectories were recorded. Successive entry into all three arms was defined as a spontaneous alteration. The percentage of spontaneous alterations ((the number of spontaneous alterations/(the total number of arm entries − 2)) × 100) was used to evaluate the spatial working memory of the mice as previously reported.^30^

#### 2.3.4 Morris water maze test

After the Y maze test, the Morris water maze (MWM) test was conducted according to a previously described procedure.^7^ In the hidden platform experiment, once a day for five continuous days, the mice were placed in the water in one of the four quadrants facing the wall of the pool and allowed to search for the underwater platform for 60 s. On the 6th day, the hidden platform was removed, and then the mice were placed in the water in the southeast quadrant facing the wall of the pool and allowed to freely swim for 60 s. The escape latency in the hidden platform experiment and number of platform crossing and target quadrant dwelling time on the 6th day were used as outcomes for evaluating spatial learning and memory ability.

### 2.4 In vivo electrophysiological recording

At day 28 after NBP treatment, in vivo electrophysiological experiments were performed to assess synaptic plasticity in the hippocampus as previously reported.^32^ A stimulation electrode and recording electrode were placed in the perforant pathway (PP) and dentate gyrus (DG), respectively. The field excitatory postsynaptic potentials (fEPSPs) were recorded at the DG electrode for 30 min as baseline by appropriate current stimulation via the PP electrode. Subsequently, a high‐frequency theta burst stimulation was given to induce LTP, which was recorded for 60 min.

### 2.5 MRI acquisition and processing

MRI data were collected using a seven Tesla vertical bore small animal MRI scanner (Bruker Biospec 94/30 USR) with a 72‐mm volume coil and a phased array mouse axial coil equipped with Paravision 360 V3.0 software after all behavioral tests were finished. The mice were anesthetized with isoflurane (4% for induction and 1.5–1.8% for maintenance) in 1.2 L/min room air mixed with 0.1 L/min oxygen. The parameters and calculations of MRI are shown in Supporting Information.

### 2.6 Laser speckle imaging

After MRI, laser speckle imaging (LSI) was conducted to evaluate CBF on the cortical surface as previously described.^33^ Briefly, perfusion images were acquired with a laser speckle contrast imager (PeriCam PSI System, Stockholm, Sweden), and PeriCam PSI HD system (Perimed, Sweden) was used to calculate the CBF values. Relative CBF was calculated using the abovementioned procedures for arterial spin labeling (ASL).

### 2.7 Evans Blue extravasation

To assess the permeability of the BBB, the Evans Blue (EB) test was carried out according to a previously reported procedure with minor modifications.^34^ EB solution (Sigma, 2%, 0.1 mL) was injected into the mice through the external jugular vein, and after 4 h, the brain tissues were removed and homogenized. Then, a spectrophotometer was used to measure the absorbance of the supernatant at a wavelength of 630 nm, and the concentration of EB in the sample was calculated according to standard solutions. The final value was calculated as the EB concentration in the sample/wet brain weight.

### 2.8 Enzyme‐Linked Immunosorbent Assay

The concentrations of proinflammatory cytokines, including TNF‐α and IL6, in brain tissue were assessed with enzyme‐linked immunosorbent assay (ELISA) according to the manufacturer's instructions (Elabscience). The brain tissue supernatant was added to the 96‐well plate, and the optical density of the sample was measured at a wavelength of 450 nm using a microplate reader (Thermo Fisher Scientific).

### 2.9 Western blot assay

Proteins were extracted from harvested hippocampal tissue with RIPA lysis buffer on ice, and the protein concentration was quantified by a BCA protein assay (Beyotime Biotechnology). Forty micrograms of proteins were loaded onto an SDS–PAGE gel for electrophoresis and then transferred to a PVDF membrane (Millipore, USA). Then, the PVDF membrane was blocked in 5% skim milk at room temperature for 1 h before being incubated with primary antibody overnight at 4°C followed by secondary antibodies for 1 h at room temperature. The signal intensity was measured using an imaging system (Tanon 5500; Tanon Science and Technology) and analyzed using ImageJ. The antibodies are shown in Supporting Information.

### 2.10 Immunofluorescence staining and imaging

The mice were perfused with 60 mL ice‐cold PBS and 4% paraformaldehyde (PFA) after anesthesia. Then, the brain tissue was removed and fixed in 4% PFA at 4°C overnight. Forty micron coronal brain slices were washed using PBST (0.3% Triton X‐100 in PBS) and then incubated with primary antibody at 4°C overnight. The slices were washed with PBST and then incubated with secondary antibodies for 1 h at room temperature. After that, the slices were washed three times, and the nuclei were stained using DAPI (1:1000; Solarbio) for 5 min. Images were captured by a confocal microscope (Olympus FV1000). The antibodies are shown in Supporting Information.

### 2.11 Statistics

Data were analyzed by SPSS 26.0 software and GraphPad Prism 8. The results were presented as mean values ± standard error of the mean (SEM). Data were tested for normality using the Kolmogorov–Smirnov test. The Student *t* test was used for two‐group comparisons in behavioral tests, except that escape latency in the MWM test was analyzed using ANOVA for repeated measurement. The one‐way ANOVA and Least Significant Difference post hoc test or Tamhane's T2 test were used in results analysis for electrophysiology, ELISA, EB test, MRI data, LSI data, and Western blotting. The null hypothesis was rejected when *p* value was <0.05.

## 3 RESULTS

### 3.1 NBP alleviated cognitive impairment and ameliorated hippocampal atrophy in BCAS model mice

**FIGURE 1:**
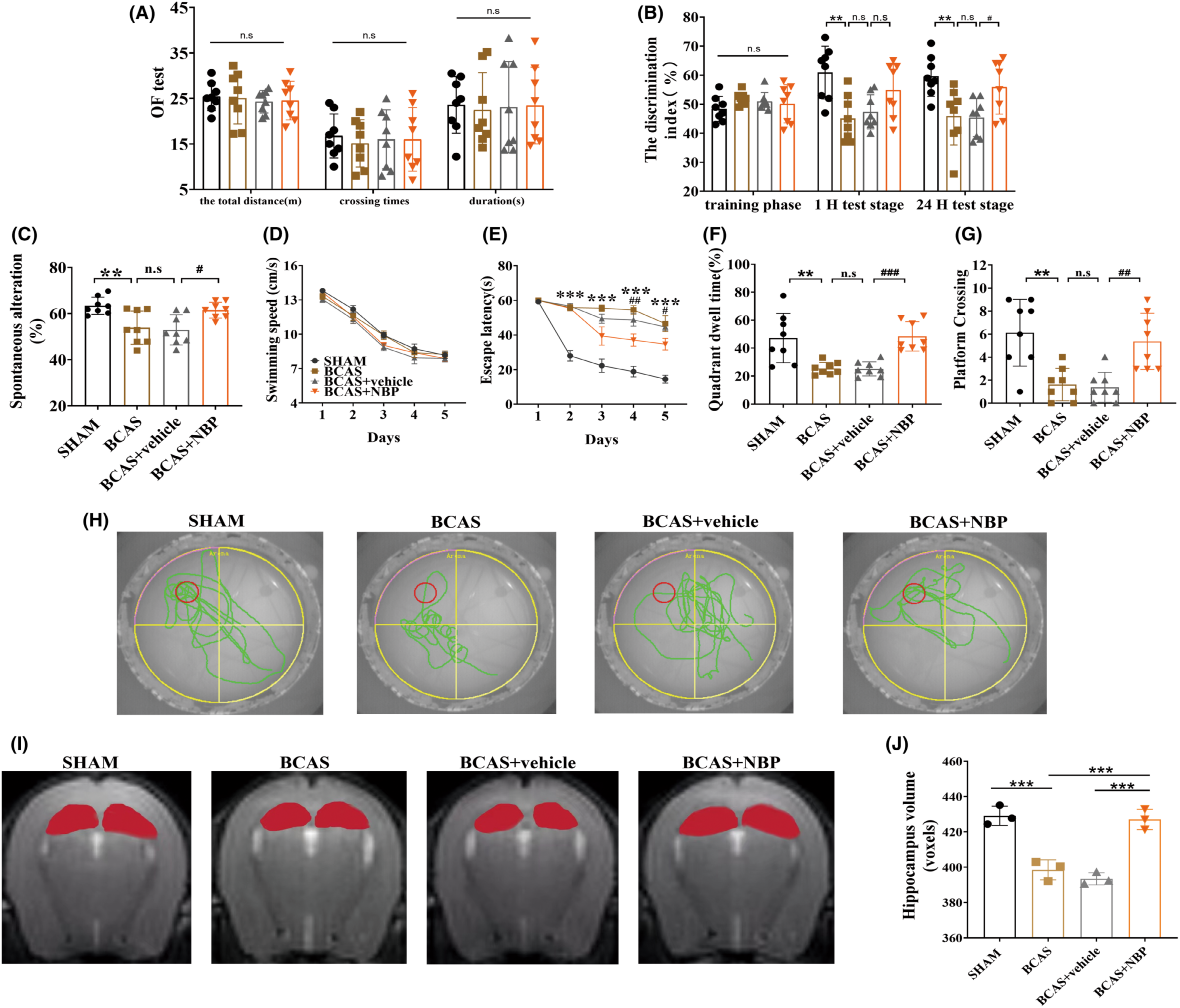
NBP alleviated cognitive impairment and ameliorated hippocampus atrophy in BCAS model mice. (A): The total distance, crossing times, and total duration spent in the central zone in the OF test (*n* = 8 in each group). (B): The DI of the mice during the training phase, the 1‐h test stage, and the 24‐h test stage in NOR test (*n* = 8 in each group). (C): The percentage of spontaneous alterations in the Y‐maze test (*n* = 8 in each group). The swimming speed (D) and the escape latency (E) at the hidden platform experiment stage; the target quadrant dwell time (F), the frequency of platform crossing (G), and the representative track (H) after removing the platform in the MWM test (*n* = 8 in each group). (I): Representative MR images of the mouse brain; the red color region indicates hippocampus. (J): Analysis of hippocampal volume in all groups (*n* = 3 in each group). BCAS, bilateral carotid artery stenosis; DI, discrimination index; MWM test, Morris water maze test; NBP, DL‐3‐n‐butylphthalide; NOR test, novel object recognition test; OF test, open‐field test. Data are shown as mean ± SEM. *p < 0.05, **p < 0.01, ***p < 0.001, SHAM vs. BCAS; #*p* < 0.05, ##*p* < 0.01, ###*p* < 0.001, BCAS+vehicle vs. BCAS+NBP, n.s means no significant difference.

The OF test results showed that there was no significant difference in total distance, crossing times, or total duration spent in the central zone among the four groups (Figure 1A), indicating that BCAS and NBP had no obvious influence on motor ability or anxiety‐like behavior.

In the NOR test, there was no significant difference in DI among any of the groups during the training phase (Figure 1B). In the 1‐h test, the BCAS group showed a lower DI than the SHAM group (Figure 1B). In the 24‐h test stage, the SHAM group explored the novel object for significantly longer than the BCAS group, and the DI was obviously increased in the BCAS + NBP group compared with the BCAS + vehicle group (Figure 1B). The DI was not significantly different between the BCAS group and the BCAS + vehicle group in either the 1‐h test or the 24‐h test.

The Y maze results showed that the BCAS group had a lower percentage of spontaneous alterations than the SHAM group, while the BCAS + NBP group had a higher percentage of spontaneous alterations than the BCAS + vehicle group (Figure 1C). There was no obvious difference between the BCAS and BCAS + vehicle groups.

In the MWM test, there was no significant difference in swimming speed among the four groups (Figure 1D). The escape latency of the BCAS group was significantly longer than that of the SHAM group on days 2–5, while the BCAS + NBP group showed a shorter escape latency than the BCAS + vehicle group on days 4–5 (Figure 1E). After removing the hidden platform, the BCAS group spent less target quadrant dwell time and made fewer platform crossing than the SHAM group. In contrast, the BCAS + NBP group spent more time in the target quadrant and made more platform crossing than the BCAS + vehicle group (Figure 1F,G). There was no significant difference in escape latency, target quadrant dwell time, or the number of platform crossings between the BCAS group and the BCAS + vehicle group. The representative track in each group is shown in Figure 1H.

Hippocampal volume was evaluated by MRI (Figure 1I). The results showed that hippocampal volume was much smaller in the BCAS group than in the SHAM group, while the BCAS + NBP group had a much larger hippocampal volume than the BCAS + vehicle group (Figure 1J). There was no difference between the BCAS group and the BCAS + vehicle group.

### 3.2 NBP attenuated synaptic plasticity impairment in BCAS model mice

**FIGURE 2:**
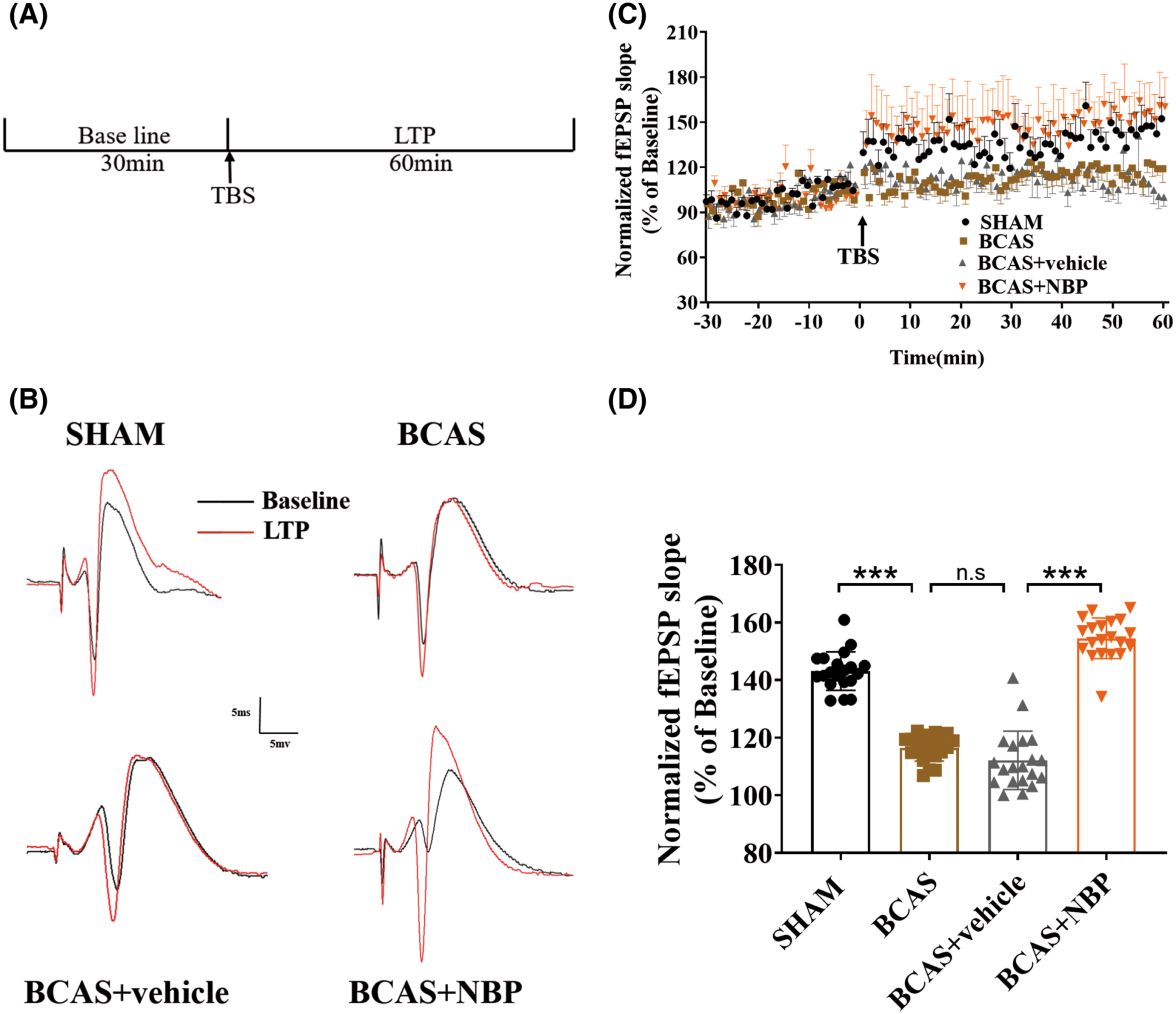
NBP attenuated synaptic plasticity impairment in BCAS model mice. (A): Timeline of in vivo electrophysiological recording. (B): Baseline period and LTP trajectory of representative fEPSPs in the four groups. (C): The fEPSP slopes changes in the PP‐DG pathway across time. In vivo electrophysiological recording of the first 30 min before receiving TBS was baseline, and the later 60 min after receiving TBS was the LTP (*n* = 7 in each group). (D): Statistical graph of mean fEPSP slope at the last 20 minutes after TBS. fEPSPs, field excitatory postsynaptic potentials; LTP, long‐term potentiation; TBS, theta burst stimulation. Data are shown as mean ± SEM, ***p < 0.001.

The timeline of the in vivo electrophysiology experiment is shown in Figure 2A. The baseline period and LTP trajectory of representative fEPSPs in the four groups are shown in Figure 2B. The fEPSP slope increased to 130%–150% compared to baseline after application of TBS in the SHAM group and BCAS + NBP group, but there was no obvious change in the BCAS group or BCAS + vehicle group (Figure 2C). Statistical analysis showed that the fEPSP slope was significantly lower in the BCAS group than in the SHAM group. The fEPSP slope was significantly higher in the BCAS + NBP group than in the BCAS + vehicle group (Figure 2D). There was no obvious difference in the fEPSP slope between the BCAS group and the BCAS + vehicle group.

### 3.3 NBP activated Akt/ERK signaling pathway in BCAS model mice

**FIGURE 3:**
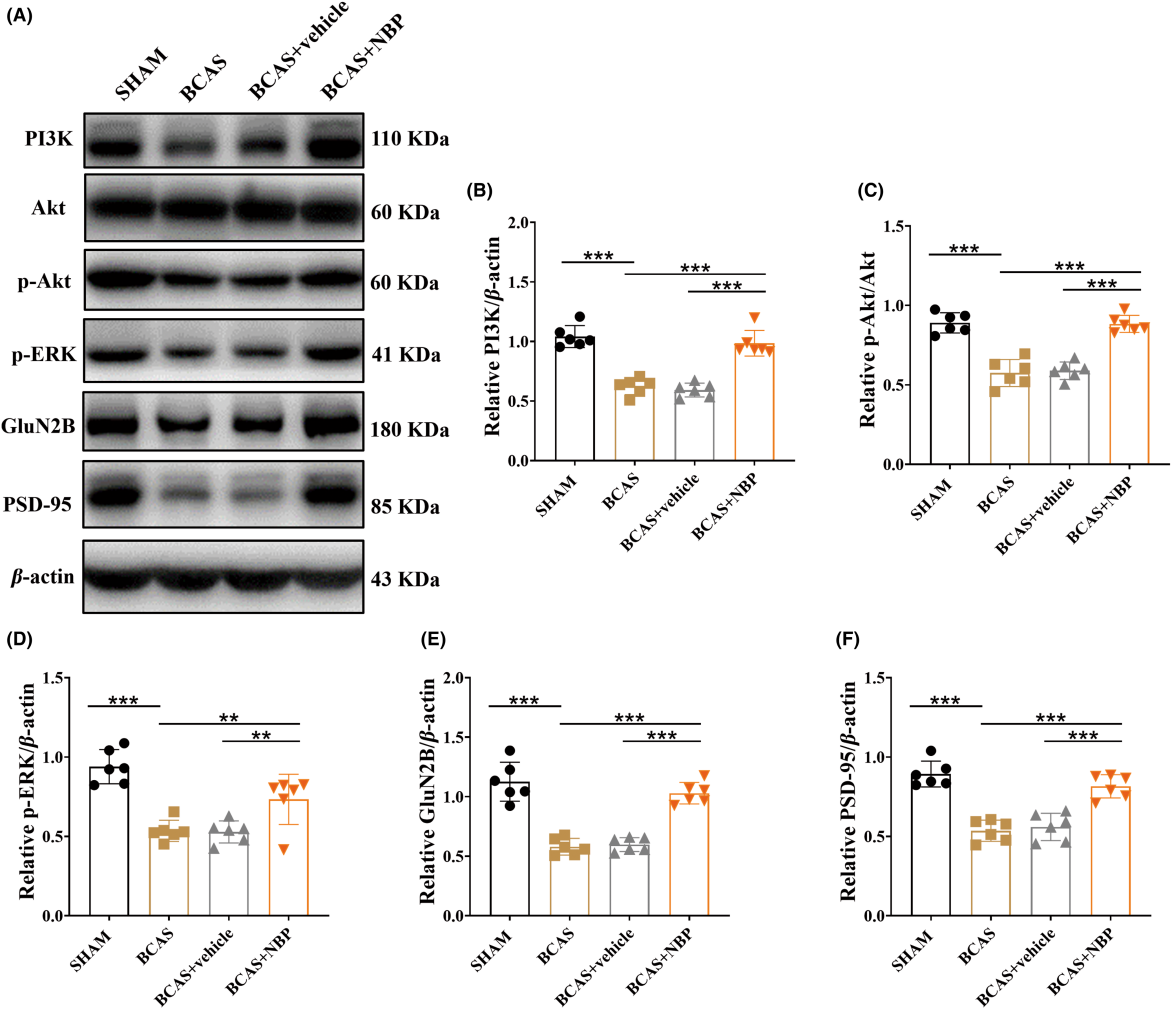
NBP activated Akt/ERK signaling pathway in BCAS model mice. (A): The representative immunoreactive bands of PI3K, Akt, p‐Akt, p‐ERK, GluN2B, PSD‐95, and β‐actin in the hippocampus. Quantitative analysis of the optical density ratio of PI3K/β‐actin (B), p‐Akt/Akt (C), p‐ERK/β‐actin (D), GluN2B/β‐actin (E) and PSD‐95/β‐actin (F), *n* = 6 in each group. Data were shown as mean ± SEM. **p < 0.01, ***p < 0.001.

The expression of proteins related to the Akt/ERK signaling pathway in the hippocampus was examined by Western blotting (Figure 3A). We found that the protein expression of PI3K, p‐Akt, and p‐ERK was significantly downregulated in the BCAS group compared with the SHAM group. However, this decrease was significantly alleviated in the BCAS + NBP group compared with the BCAS + vehicle group (Figure 3B–D). The expression of GluN2B and PSD‐95, as downstream effectors of the Akt/ERK signaling pathway, was obviously reduced in the BCAS group compared with the SHAM group; in contrast, their expression was higher in the BCAS + NBP group than in the BCAS + vehicle group (Figure 3E,F). There were no significant differences in the expression of any of these proteins between the BCAS group and the BCAS + vehicle group.

### 3.4 NBP increased the CBF and preserved white matter integrity in BCAS model mice

**FIGURE 4:**
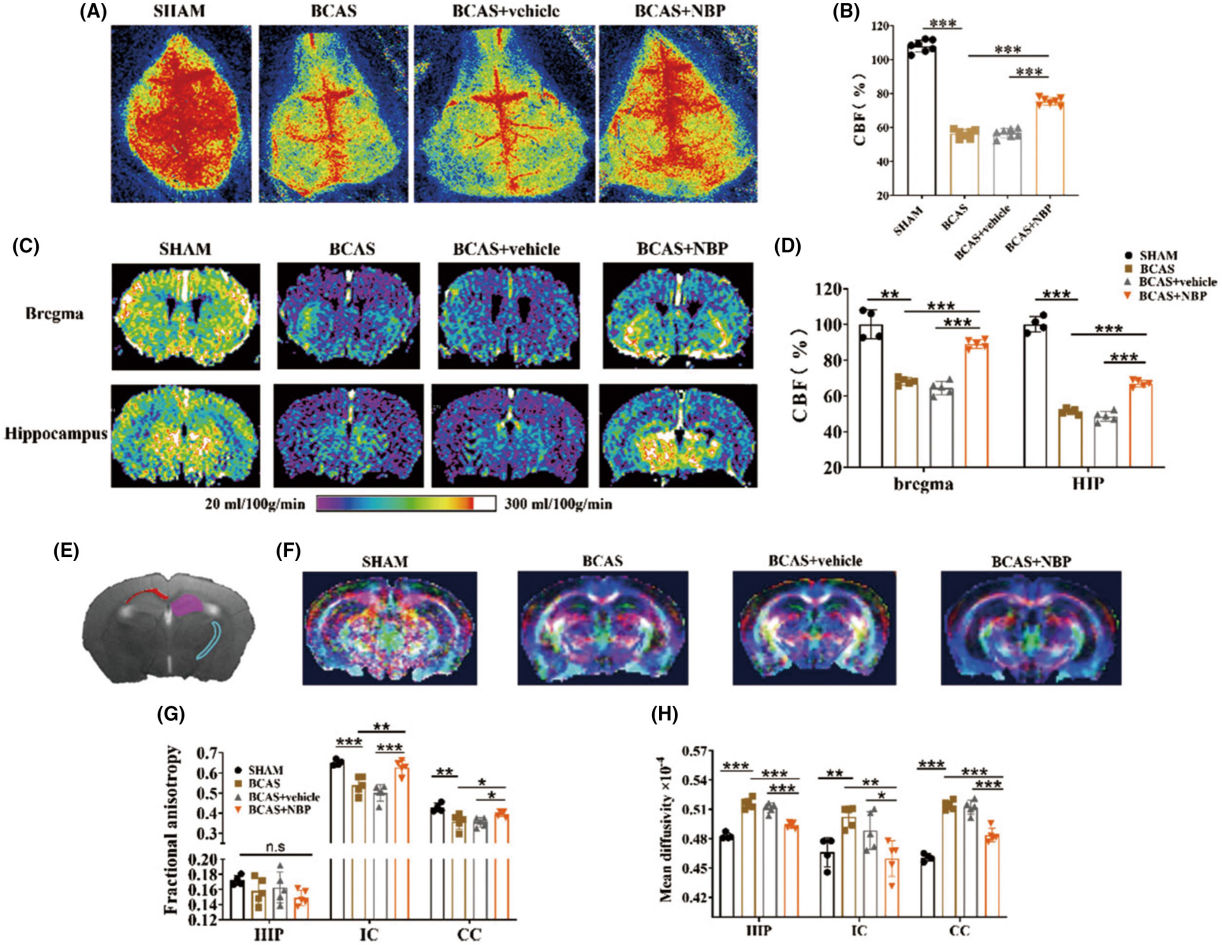
NBP increased the CBF and preserved white matter integrity in BCAS model mice. (A): The representative cortical CBF images obtained from LSI. (B): Quantitative analysis of CBF measured with LSI in different groups (*n* = 7 in each group). (C): The representative multislice coronal CBF images acquired from ASL at the bregma and hippocampus. (D): Quantitative analysis of CBF measured with ASL in different groups (*n* = 4 in the SHAM group, *n* = 5 in the other three groups). (E): Regions of interest analysis on T2‐weighted volume, purple color stands for HIP, blue color stands for IC, and red color stands for CC. (F): Representative DEC maps for DTI analysis in four groups. (G): Quantitative analysis of mean FA in HIP, IC, and CC. (H): Quantitative analysis of MD values in HIP, IC, and CC (*n* = 4 in the SHAM group, *n* = 5 in the other three groups). ASL, arterial spin labeling; CBF, cerebral blood flow; CC, corpus callosum; DEC, directionally encoded color; DTI, diffusion tensor imaging; FA, fractional anisotropy; HIP, hippocampus; IC, internal capsule; LSI, laser speckle imaging; MD, mean diffusivity. Data are shown as mean ± SEM, *p < 0.05, **p < 0.01, ***p < 0.001, n.s means no significant difference.

CBF on the cortical surface, which was measured by LSI, was sharply decreased in the BCAS group compared with the SHAM group, while CBF was significantly increased in the BCAS + NBP group compared with the BCAS + vehicle group (Figure 4A,B). ASL MRI was performed to explore CBF in deep brain structures at the levels of bregma and the hippocampus. Notably, CBF at the levels of bregma and the hippocampus was lower in the BCAS group than in the SHAM group. However, CBF was significantly increased in the BCAS + NBP group compared with the BCAS + vehicle group (Figure 4C,D). There was no significant difference in CBF measured with LSI or ASL MRI between the BCAS group and the BCAS + vehicle group.

Diffusion tensor imaging (DTI) MRI, which can be used to assess white matter demyelination due to ischemia, was used to measure the integrity of white matter tracts. The hippocampus, internal capsule (IC), and corpus callosum (CC) were selected as the regions of interest (Figure 4E). Representative DEC maps in the four groups are shown in Figure 4F. DTI metrics and analysis of white matter tracts showed that the mean fractional anisotropy (FA) values were lower in the IC and the CC and the mean mean diffusivity (MD) values were higher in the hippocampus, the IC, and the CC in the BCAS group than in the SHAM group. Compared with the BCAS+vehicle group, the BCAS + NBP group showed significant increases in mean FA values in the IC and the CC and decreases in mean MD values in all three regions (Figure 4G,H).

### 3.5 NBP attenuated brain inflammation and reduced the BBB leakage in BCAS model mice

**FIGURE 5:**
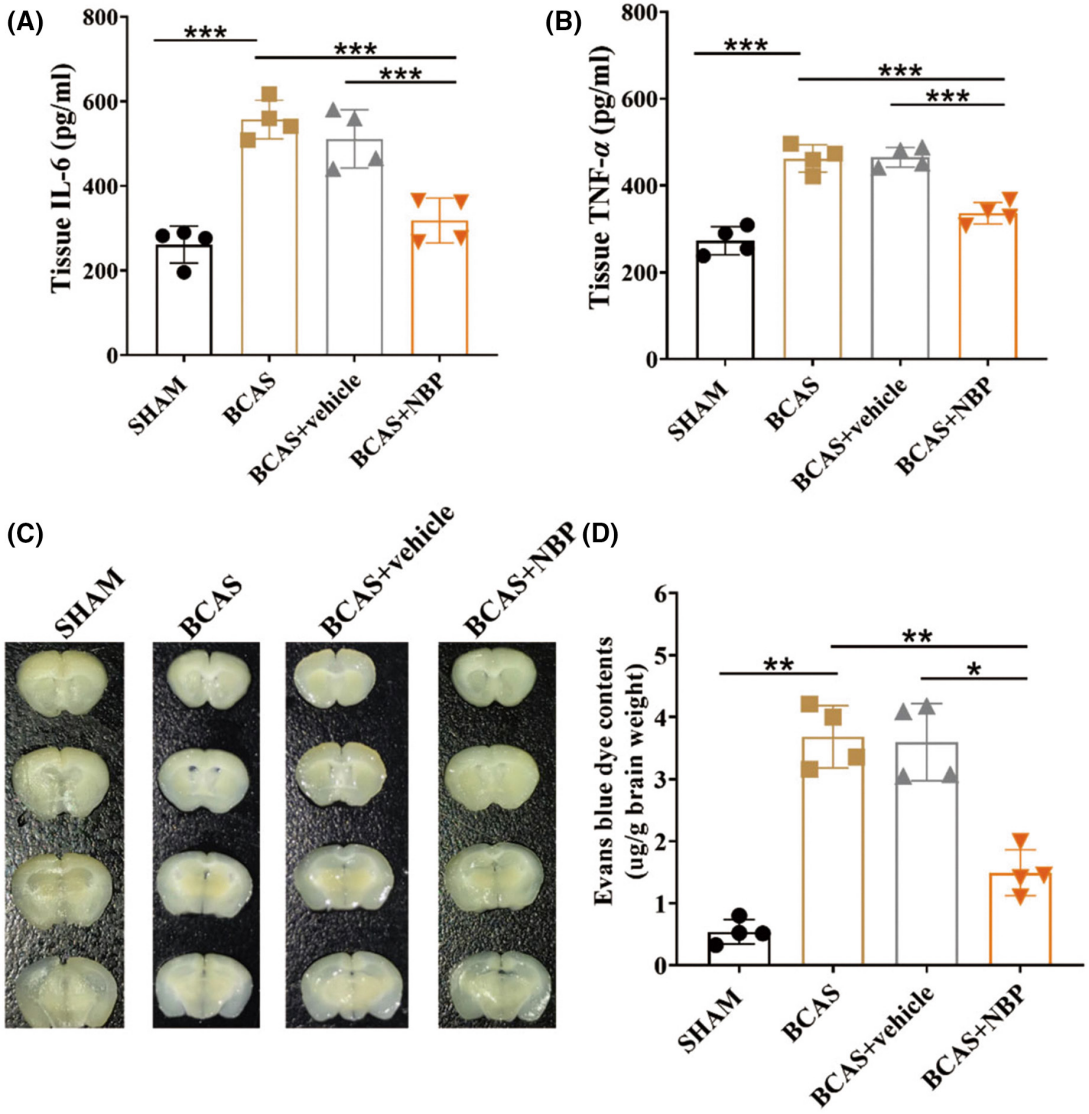
NBP attenuated brain inflammation and reduced the BBB leakage in BCAS model mice. The concentrations of inflammatory cytokines IL‐6 (A) and TNF‐α (B) in the whole brain tissues were detected by ELISA (*n* = 4 in each group). (C): Representative images of Evans blue extravasation. (D): Quantitative analysis of Evans blue extravasation (*n* = 4 in each group). Data are shown as mean ± SEM. *p < 0.05, **p < 0.01, ***p < 0.001.

The concentrations of inflammatory cytokines (IL‐6 and TNF‐α) in brain tissue were higher in the BCAS model mice than in the SHAM mice and were significantly reduced in the BCAS + NBP group compared with the BCAS + vehicle group (Figure 5A,B). Moreover, the rate of BBB leakage was assessed through the EB extravasation assay. The concentrations of EB in brain tissue were higher in the BCAS group than in the SHAM group and were relatively reduced in the BCAS + NBP group compared with the BCAS + vehicle group (Figure 5C,D).

### 3.6 NBP inhibited the HIF‐1α/MMP signaling pathway and up‐regulated the expression of TJ proteins in BCAS model mice

**FIGURE 6:**
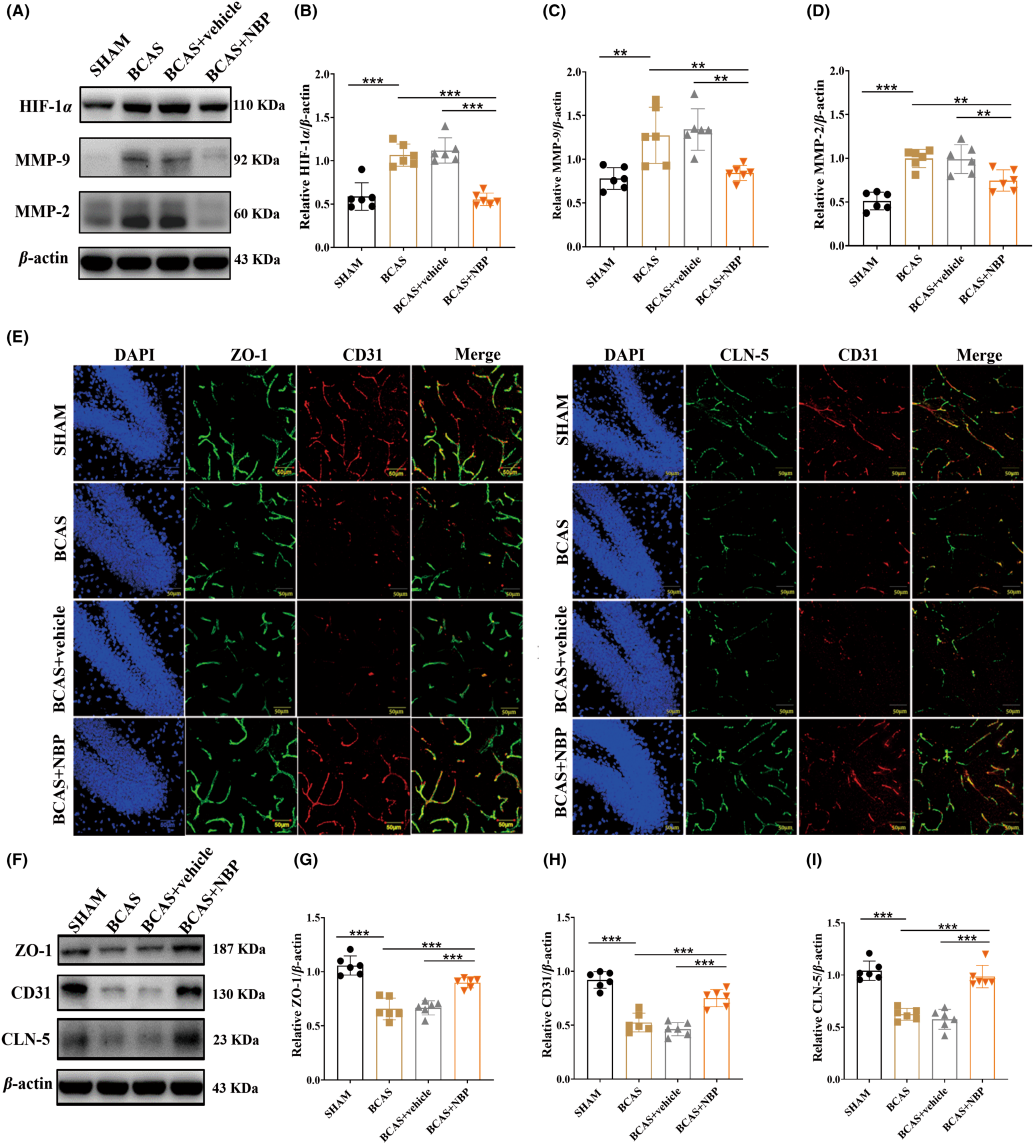
NBP inhibited the HIF‐1α/MMP signaling pathway and up‐regulated the expression of TJ proteins in BCAS model mice. (A): The representative immunoreactive bands of HIF‐1α, MMP‐9, and MMP‐2 and β‐actin in the hippocampus. Quantitative analysis of the optical density ratio of HIF‐1α (B), MMP‐9 (C) and MMP‐2 (D) in the hippocampus (*n* = 6 in each group). (E): Representative images of ZO‐1 colabeled with CD31 and CLN‐5 colabeled with CD31 in the hippocampus, scale bar: 50 μm (n = 3 in each group). (F): The representative immunoreactive bands of ZO‐1, CD31, CLN‐5 and β‐actin in the hippocampus. Quantitative analysis of the optical density ratio of ZO‐1 (G), CD31 (H) and CLN‐5 (I) in the hippocampus (*n* = 6 in each group). Data are shown as mean ± SEM. **p < 0.01, ***p < 0.001.

Western blotting showed that the expression of HIF‐1α, MMP‐9, and MMP‐2 was upregulated in the BCAS group compared with the SHAM group and was obviously decreased in the BCAS + NBP group compared to the BCAS + vehicle group (Figure 6A–D). Moreover, the levels of proteins downstream of the HIF‐1α/MMP pathway, including ZO‐1, CLN‐5 and CD31, which are indispensable for maintaining the structural integrity of the BBB, were measured by Western blotting and IF. Both assays showed that the expression levels of ZO‐1, CLN‐5, and CD31 were significantly decreased in the BCAS group compared to the SHAM group and that this decrease could be alleviated after treatment with NBP (Figure 6E–I).

## 4 DISCUSSION

This study showed that NBP alleviated cognitive impairment in a mouse model of VaD induced by BCAS. First, our results demonstrated that NBP attenuated hippocampal LTP impairment and ameliorated hippocampal atrophy measured with MRI, which are crucial for learning and memory. Moreover, we found that NBP may activate the Akt/ERK signaling pathway, which is important for synaptic plasticity. Finally, BBB leakage was reduced, and the HIF‐1α/MMP signaling pathway may be inhibited by NBP in this study. Therefore, our study demonstrated that NBP exerted beneficial effects on cognition in VaD through multiple mechanisms.

The hippocampus is an important part of the limbic system, which is associated with cognitive function. Hippocampal atrophy is a key pathological feature of AD,^35^ but studies investigating the change in hippocampal volume in VaD models are limited. To our knowledge, the current study was the first to measure the hippocampal volume of mice subjected to BCAS by MRI. The results showed that the hippocampal volume was less in the BCAS group than in the SHAM group. In a previous study, histological assessment revealed obvious hippocampal atrophy in mice at 8 months after BCAS.^36^ This finding indicates that hippocampal damage contributes to or is associated with the pathogenesis of VaD. However, our results showed that treatment with NBP could ameliorate hippocampal atrophy, in line with previous studies using histological assays to measure hippocampal volume.^37^, ^38^, ^39^ Moreover, NBP markedly enhanced hippocampal LTP from the PP to the DG. This finding is consistent with the results of a previous study in which 2VO was used to establish a VaD model.^28^ Since LTP is an in vivo measurement of synaptic plasticity, all these findings suggested that NBP could protect against pathological changes in hippocampal structure and function resulting from chronic ischemia.

We further found that NBP activated the Akt/ERK signaling pathway and upregulated the expression of synapse‐related proteins. A large number of studies have confirmed that Akt and ERK are involved in the regulation of synaptic function. It was found that activation of the PI3K/Akt signaling pathway increased the dendritic spine density and promoted synaptic plasticity in middle cerebral artery occlusion/reperfusion injury rats.^40^ Our results are inconsistent with previous findings that NBP ameliorated cognitive decline by promoting the PI3K/Akt signaling pathway in *db/db* mice, a model of type‐2 diabetes.^41^ Other studies also demonstrated that NBP treatment attenuated depression‐like behaviors and ameliorated spatial learning and memory impairment by upregulating the expression of p‐ERK and p‐Akt in rats exposed to chronic suspension stress and APP/PS1 transgenic AD mice.^42^, ^43^ It was further demonstrated that the effect of NBP on reducing hippocampal neuronal apoptosis could be reduced entirely by inhibiting p‐Akt and p‐ERK, in oxygen–glucose deprivation cell model.^38^ Taken together, these results suggest that the effects of NBP in enhancing hippocampal LTP and ameliorating cognitive decline may involve regulation of the Akt/ERK signaling pathway and its downstream synaptic mediators GluN2B and PSD‐95.

Hypoperfusion is an important factor contributing to the pathogenesis and pathophysiology of neurodegenerative and cerebrovascular diseases, particularly VaD. In the present study, we found that NBP increased CBF in both cortical and deep brain structures in mice subjected to BCAS, which is consistent with previous findings in a VaD model.^27^, ^44^ It was demonstrated that improvements in endothelial function, rescue of neurovascular coupling responses, and increases in CBF likely contribute to improved cortical function in aged mice.^45^ Therefore, NBP may improve cognitive function and synaptic plasticity in VaD by increasing CBF. Moreover, MRI demonstrated that NBP treatment increased the mean FA values in the IC and CC in mice subjected to BCAS, suggesting that NBP protects white matter integrity under chronic hypoperfusion. Previous studies have found that a decrease in the FA value is a highly sensitive indicator of white matter microstructural damage, which linearly correlates with worse performance in the Y maze test in mice subjected to BCAS.^46^, ^47^ Thus, the ability of NBP to improve cognition could be attributed to its protection of white matter.

In terms of BBB permeability, our results showed higher concentrations of EB and the proinflammatory factors TNF‐α and IL‐6 in the brain tissues of mice subjected to BCAS. Concomitantly, the expression of HIF‐1α, which can be upregulated under hypoperfusion and hypoxia and is associated with activation of the neuroinflammatory response and its downstream proteins MMP‐2 and MMP‐9, was increased in the hippocampi of mice subjected to BCAS. Previous studies have demonstrated that HIF‐1α is associated with elevation of MMP levels in vivo and vitro models, which played an important role in BBB dysfunction.^20^, ^24^, ^48^ In a previous study, it was found that TNF‐α could induce the expression of HIF‐1α at the mRNA and protein levels and then increase the expression of MMP‐9, which further degenerated tight junctions and increased endothelial permeability.^49^ In another study, pretreatment with YC‐1, an HIF‐1α inhibitor, significantly downregulated MMP‐2 expression and alleviated BBB damage in middle cerebral artery occlusion model rats.^50^

Molecular transport across the BBB is precisely regulated by TJ proteins, including ZO1, CLN‐5, and junction adhesion molecules, which are important elements of the junctional complexes.^51^ In the present study, the protein expression of ZO1, CLN‐5, and CD31 was significantly decreased in the hippocampi of mice subjected to BCAS. Combined with previous findings, these results indicated that insufficient CBF increases BBB leakage, the infiltration of proinflammatory factors from the blood into the brain, and activation of the HIF‐1α/MMP signaling pathway, subsequently downregulating TJ protein expression and eventually leading to neurovascular dysfunction in mice subjected to BCAS.^52^, ^53^ In the present study, we found that NBP treatment reduced the expression of HIF‐1α and its downstream proteins, such as MMP‐2 and MMP‐9. A previous study claimed that NBP protected BBB integrity and attenuated brain injury in the acute phase of ischemic stroke by decreasing MMP‐9 enzyme activity.^54^ In addition, it was demonstrated that NBP promoted angiogenesis and cognitive function in chronic hypoxia and hypoperfusion model mice by mediating the HIF‐1α signaling pathway.^55^, ^56^ Taken together, we suggested that NBP may inhibit BBB leakage and inflammation by inhibiting the HIF‐1α/MMP signaling pathway in mice subjected to BCAS.

There are some limitations in this study. First, the BCAS model recapitulates the pathogenesis of VaD caused by hypoperfusion but not that of VaD caused by aging or common vascular risk factors, such as hypertension, diabetes mellitus, and hypercholesterolemia, in humans. Second, we only examined pathological and pathophysiological changes at 6 weeks after BCAS. The long‐term effect and the optimal treatment course of NBP were not explored in this study. Moreover, only one dose was used in the current study based on previous evidence from VaD model mice, and the therapeutic effects of NBP at different doses after BCAS are worthy of investigation. Finally, we did not validate the effects of NBP on cognitive and BBB protection through mediating the Akt/ERK and HIF‐1α/MMP signaling pathways by further inhibiting Akt/ERK or activating HIF‐1α.

In conclusion, our results demonstrated that BCAS could cause cognitive impairment and a series of pathological changes, including impairment of synaptic plasticity and BBB destruction, which partly mimic the pathological changes in patients with VaD. Furthermore, NBP may enhance hippocampal LTP and improve cerebral perfusion and BBB integrity by mediating the Akt/ERK and HIF‐1α/MMP signaling pathways, respectively, suggesting that it exerts a promising therapeutic effect on VaD treatment through multiple mechanisms.

## AUTHOR CONTRIBUTIONS

Ping Che and Nan Zhang conceived and designed the experiments. Ping Che, Juan Zhang, Mingqian Yu, Ping Tang, and Yanhui Wang performed the experiments. Ping Che analyzed the data and wrote the manuscript. Aolei Lin and Jing Xu gave crucial comments for the manuscript. Nan Zhang formulated the research questions and revised the manuscript.

## CONFLICT OF INTEREST STATEMENT

The authors declare that they have no conflict of interest.

## Supporting information


Appendix S1

## Data Availability

The original contributions presented in the study are included in the article/Supporting Information; further inquiries can be directed to the corresponding author.
