# Supplementary material for: Dl‐3‐n‐butylphthalide promotes synaptic plasticity by activating the Akt/ERK signaling pathway and reduces the blood–brain barrier leakage by inhibiting the HIF‐1α/MMP signaling pathway in vascular dementia model mice
Source: CNS Neurosci Ther. 2023 Feb 8;29(5):1392–404. doi: 10.1111/cns.14112 (PMC10068471; doi:10.1111/cns.14112)
Supplement: Supplementary file 1 — Appendix S1 [file CNS-29-1392-s001.docx]

**
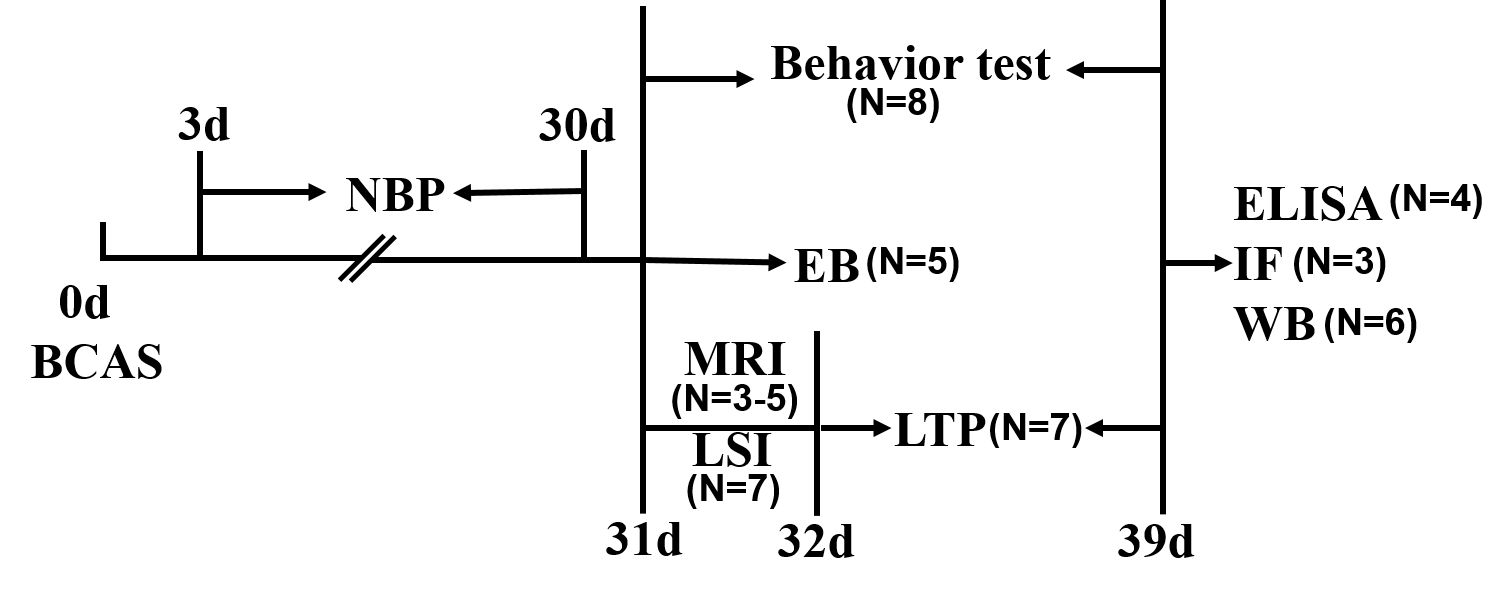
**

**FIGURE 1 The timeline of all experiments**

**METHODS**

**1. MRI acquisition and processing**

MRI data were collected using a 7 Tesla vertical bore small animal MRI scanner (Bruker Biospec 94/30 USR) with a 72 mm volume coil and a phased array mouse axial coil equipped with Paravision 360 V3.0 software after all behavioral tests were finished. The mice were anesthetized with isoflurane (4% for induction and 1.5–1.8% for maintenance) in 1.2 L/min room air mixed with 0.1 L/min oxygen. Body temperature, heart rate and respiratory rate were monitored. T2-weighted images were obtained by a rapid acquisition relaxation enhancement sequence with the following parameters: repetition time (TR) = 2500 ms, echo time (TE) = 33 ms, rare factor = 8, field of vision (FOV) = 15 × 5 mm, matrix size = 256 × 256, slice thickness = 0.4 mm, scanning time: 1 min 20 s. 3D-T2 images were obtained with the following parameters: TR = 1800 ms, TE = 36.38 ms, rare factor = 12, FOV = 15 × 15 × 7.5 mm, matrix size = 100 × 100 × 50, slice thickness = 7.5 mm, scanning time: 13 min 12 s. Cerebral blood flow (CBF) in coronal slices was measured using arterial spin labeling (ASL), a flow-sensitive alternating inversion recovery technique, with the following parameters: TR = 10000 ms, TE = 20.11 ms, FOV = 15 × 15 mm, matrix size = 128 × 128, slice thickness = 1 mm, scanning time: 5 min 48 s. Diffusion tensor imaging (DTI) was performed using a multislice spin echo sequence with the following parameters: b value = 2000 and 1000 s/mm2; TR/TE = 3000/35 ms, 30 diffusion directions, slice thickness = 0.4 mm, FOV = 19.2 × 19.2 mm, scanning time: 13 min.

The hippocampus was outlined on 3D-T2 images using MRIcron software, and the hippocampal volume was calculated with MATLAB. ASL images (bregma level and hippocampus level) were reconstructed by using ParaVison 360 V3.0 software (Bruker, Pharmascan), and the mean CBF in two coronal slices was calculated. CBF value from the sham group was used as a reference, and for each mouse, the relative CBF was calculated as a percentage of that reference value. Directionally encoded color (DEC), fractional anisotropy (FA) and mean diffusivity (MD) images obtained by DTI were reconstructed using ParaVison 360 V3.0 software (Bruker, Pharmascan). Then, the FA and MD values in regions of interest, including the hippocampus (HIP), internal capsule (IC) and corpus callosum (CC), which were drawn by a blinded manner on T2-weighted images, were calculated.

**2. Western blot assay**

Proteins were extracted from harvested hippocampal tissue with RIPA lysis buffer on ice, and the protein concentration was quantified by a BCA protein assay (Beyotime Biotechnology, China). Forty micrograms of protein was loaded onto an SDS‒PAGE gel for electrophoresis and then transferred to a PVDF membrane (Millipore, USA). Then, the PVDF membrane was blocked in 5% skim milk at room temperature for 1 hour before being incubated with primary antibody overnight at 4°C followed by HRP-conjugated anti-rabbit IgG (H + L) (1:5000, Promega) and HRP-conjugated anti-mouse IgG (H + L) HRP conjugate (1:5000, Promega) secondary antibodies for 1 hour at room temperature. The signal intensity was measured using an imaging system (Tanon 5500, Tanon Science and Technology) and analyzed using ImageJ. The following primary antibodies were used for the Western blot analysis: β-actin (1:3000, Sangon Biotech, D110001), MMP-2 (1:500, Santa Cruz, sc-13594), MMP-9 (1:500, Santa Cruz, sc-393859), CD31 (1:500, Santa Cruz, sc-376764), ZO-1 (1:1000, Abcam, ab96587), claudin-5 (CLN-5, 1:2000, Invitrogen, 4C3C2), PSD-95 (1:1000, Abcam, ab2723), GluN2B (1:1000, Abcam, ab254356), PI3K (1:1000, Abcam, ab109006), Akt (1:1000, Abcam, ab179463), p-Akt (1:1000, Abcam, ab192623), and p-ERK (1:1000, Abcam, ab201015).

**3. Immunofluorescence staining and imaging**

The mice were perfused with 60 ml ice-cold PBS and 4% paraformaldehyde (PFA) after anesthesia. Then, the brain tissue was removed and fixed in 4% PFA at 4°C overnight. Forty micron coronal brain slices were washed using PBST (0.3% Triton X-100 in PBS) and then incubated with primary antibody at 4°C overnight. The slices were washed with PBST and then incubated with secondary antibodies (Alexa 488-conjugated goat anti-mouse IgG, Alexa 594-conjugated goat anti-rat IgG and Alexa 488-conjugated goat anti-rabbit IgG) for 1 hour at room temperature. After that, the slices were washed three times, and the nuclei were stained using DAPI (1:1000, Solarbio, China) for 5 min. Images were captured by a confocal microscope (Olympus FV1000, Japan). The primary antibodies were as follows: CD31 (1:200, Santa Cruz, sc-18916), ZO-1 (1:500, Abcam, ab96587), and CLN-5 (1:500, Invitrogen, 4C3C2).
